# Non-Invasive Mapping of the Neuronal Networks of Language

**DOI:** 10.3390/brainsci13101457

**Published:** 2023-10-13

**Authors:** Andrew C. Papanicolaou

**Affiliations:** Department of Pediatrics, Division of Pediatric Neurology, College of Medicine, University of Tennessee Health Science Center, Memphis, TN 38013, USA; andreascpapanicolaou@gmail.com; Tel.: +1-(901)-287-5207

**Keywords:** language, networks, non-invasive brain mapping, presurgical functional mapping

## Abstract

This review consists of three main sections. In the first, the Introduction, the main theories of the neuronal mediation of linguistic operations, derived mostly from studies of the effects of focal lesions on linguistic performance, are summarized. These models furnish the conceptual framework on which the design of subsequent functional neuroimaging investigations is based. In the second section, the methods of functional neuroimaging, especially those of functional Magnetic Resonance Imaging (fMRI) and of Magnetoencephalography (MEG), are detailed along with the specific activation tasks employed in presurgical functional mapping. The reliability of these non-invasive methods and their validity, judged against the results of the invasive methods, namely, the “Wada” procedure and Cortical Stimulation Mapping (CSM), is assessed and their use in presurgical mapping is justified. In the third and final section, the applications of fMRI and MEG in basic research are surveyed in the following six sub-sections, each dealing with the assessment of the neuronal networks for (1) the acoustic and phonological, (2) for semantic, (3) for syntactic, (4) for prosodic operations, (5) for sign language and (6) for the operations of reading and the mechanisms of dyslexia.

## 1. Introduction

Mapping the neuronal networks of language has been a popular scientific pursuit since the functional neuroimaging methods became adequate for the task. In addition to its theoretical value, such mapping is of considerable practical utility as an adjunct to or a replacement of the traditional invasive presurgical mapping techniques, namely the Intracarotid Sodium Amytal test (or the Wada procedure) for assessing lateralization of the language networks and the direct cortical stimulation mapping (CSM) method for localizing parts of such networks in the cortex of the language dominant hemisphere (for reviews, see [1,2,3,4]).

Language, conceived as a function, is not a monolithic entity but consists of at least four interrelated yet distinct subsidiary operations, each apparently associated with its own neuronal network. These are the acoustic, the phonological, the semantic and the syntactic operations. The first analyzes the acoustic features of speech sounds; the second those features that are specific to human speech; and the third is assumed to invest these signals with meaning in ways that remain largely conjectural. Finally, the fourth provides the means for arranging words in serial order so as to constitute grammatically correct sentences but also for arranging phonemes in the requisite serial order for the construction (and comprehension) of words.

It is nearly certain that not all four of these operations are lateralized. The acoustic processing of both speech and non-speech stimuli is generally believed to be mediated by neuronal networks in the auditory cortex in Heschl’s Gyrus (HG) in the mid-portion of the superior temporal gyrus (STG) in the left and in the right hemispheres. In contrast, speech production (which includes the temporal arrangement of articulatory gestures for producing words as well as the arrangement of words for producing sentences) is said to involve the Inferior Frontal Gyrus (IFG), comprising the pars opercularis and pars triangularis, of the left hemisphere only. There is also considerable agreement that the IFG may be necessary for the comprehension of sentences, although its contribution may not be necessary for the comprehension of isolated words [5].

Beyond these points of general agreement, most other hypotheses regarding cortical organization for language vary among theorists. According to the dominant model that has emerged over the years, mostly on the basis of focal brain lesion studies, phonological processing requires the contribution of the posterior part of STG (pSTG) of only the left hemisphere [6,7,8,9]. But this view has been challenged, partly on the basis of results of some focal lesion studies [10,11,12,13,14] and partly on some functional neuroimaging data [15,16]. Instead, a “dual-route” model has been proposed, according to which the perception of speech sounds engages networks in both the left and the right pSTG but also of the anterior STG, e.g., [8,17,18,19,20,21,22].

The situation with respect to the lateralization of semantic operations is even less settled. According to some investigators, semantic operations are mediated by networks in the region that encompasses the pSTG, a portion of the Middle Temporal Gyrus (MTG) and the Angular and Supramarginal gyri (AG and SMG, respectively) of the left cerebral hemisphere, that is, the classical “Wernicke’s area.” This view, which has been widely supported by clinical evidence that lesions in this area disrupt the comprehension of both words and sentences [6,23,24,25,26,27,28], has been challenged by dementia data [29,30,31,32,33,34,35] as well as focal lesion data [36]. These data indicate that whereas Wernicke’s area may appear to be necessary for both phonological and semantic processes, it is, in fact, necessary for phonological analysis only. This analysis is said to result in the emergence of the word-forms (i.e., activation patterns coding the phonological aspect of words)—see [37,38]. Nevertheless, in the context of this view, its disruption would be expected to interfere with not only phonological analysis but also with word and sentence comprehension, because the latter presuppose phonological analysis and not because the area mediates semantic operations. Semantic operations, in this alternative view, are mediated by the left Anterior Temporal Lobe (ATL) instead. However, the precise role of the ATL is not at all clear. According to one hypothesis [5], the ATL is necessary for mediating the activation of semantic circuits distributed throughout the cortex on the basis of “word form”-related input that it receives from Wernicke’s region, or for activating word-form circuits on the basis of input from semantic circuits, as in the case of object naming tasks [35,36]. Therefore, according to this alternative to the dominant model, the disruption of the left ATL by electrical stimulation would be expected to disrupt word comprehension (consequently, also, sentence comprehension), as well as object naming. But the results of a cortical stimulation mapping (CSM) study [39] have pointed to the conclusion that the left fusiform gyrus (rather than the left ATL) is necessary for word and sentence comprehension. On the basis of yet another lesion study [40], it has also been proposed that comprehension of word meaning is mediated by the inferior temporal region and the ATL bilaterally. These data lead to the expectation that, during word comprehension tasks, all these areas, rather than only the left ATL or the left Wernicke’s region, should show increased activation [21].

It is in the settlement of this largely unsettled terrain of mixed facts and conjectures that functional neuroimaging is attempting to make its contribution. In the sections that follow, we will briefly review the nature of the functional neuroimaging methods employed in that capacity, and we will comment on the justification of their use by pointing out the compatibility of their results with those of the traditional invasive brain mapping methods. In a final section, the most notable results of the application of these methods to the study of linguistic networks will be summarized.

## 2. The Methods of Non-Invasive Mapping

### 2.1. Methods

There are four types of neurophysiological events that are typically captured in functional images, each associated with a different kind of electromagnetic signal. Three of the four types of brain events imaged are aspects of what is referred to as brain baseline “activity” and stimulus or task-specific “activation”. These are, first, electrochemical signaling among neurons, which is imaged through the method of magnetoencephalography (MEG). Second, metabolic activity rates in sets of neurons. These are imaged by means of Positron Emission Tomography (PET). Third, blood flow rates supplying glucose and oxygen to these sets of neurons, imaged through PET but, most commonly, through functional Magnetic Resonance Imaging (fMRI). These three aspects of activity and activation are interrelated: local rates of signaling, specific to each brain structure at rest and during its engagement in behavioral and cognitive tasks, determine to a large extent the rates at which these structures utilize glucose and oxygen. These local metabolic rates, in turn, determine, along with other factors, the rates of local blood flow. This being the case, maps of brain activity and activation representing rates of signaling or of metabolism or of blood flow, recorded under the same circumstances, are expected to be quite similar, especially in normal individuals, if not also in patients sustaining vascular or other brain lesions. The fourth type of brain process is a prerequisite of brain activity and activation, and changes sufficiently slowly so as to qualify as a time-invariant; that is, a structural aspect of brain physiology. It consists of the distributions of receptors for particular classes of neurotransmitters throughout the brain, and is imaged using PET.

The baseline activity and the activation patterns imaged represent three basic types of entities: first, the “functional networks” that embody the mechanisms of particular functions or subsidiary operations. They are obtained in activation experiments and are typically captured using fMRI (there are many more fMRI scanners than all MEG and PET systems combined), less frequently with MEG, occasionally using blood flow PET and, rarely, via metabolic PET. Second, the activation patterns represent signals putatively specific to particular “products” of functions, such as different classes of behavior, i.e., percepts, sensations, thoughts or sentiments. Third, the visualized patterns of resting activity may represent features of physiology specific to different diagnostic categories, personality traits, demographic categories (i.e., age, gender) and relatively long-lasting “states” of the subject (e.g., vigilance, craving, anger). Here, the methods of choice are PET (which could capture category-specific receptor distributions for particular neurotransmitters) and metabolic PET, although fMRI and MEG have been used for that purpose.

### 2.2. Tasks

The activation tasks used fall into two main categories: those of production and of perception of either phonemes, morphemes or whole sentences presented either auditorily or visually (reading). In detail, the tasks vary widely depending on the specific aims of particular neuroimaging studies. However, there is a more-or-less standardized set of tasks that have been adopted in clinical settings of presurgical functional mapping represented by the following typical examples used with MEG and fMRI.

The most common task for expressive language mapping with fMRI involves the covert production of words to visual cues (for detailed discussion, see also [41,42]). Several variants of this task are in use. In verb generation tasks, the patient is asked to silently produce action verbs (e.g., “cut, slice”) in response to a printed noun (e.g., “knife”) or to produce nouns that belong to a particular semantic category. Activation maps obtained during these tasks are compared to activation maps obtained during rest or during the passive viewing of meaningless letter strings. Alternative tasks often used for expressive language mapping involve covert object naming and sentence completion (e.g., [43]). A typical object naming task consists of a standard block design alternating between stimulus and rest, during which the patient is presented with line drawings of living and inanimate objects [44] with the explicit instruction to covertly name the object upon presentation.

Receptive language mapping with fMRI generally follows the protocol described by Binder et al. [45] involving a blocked semantic/tone decision task (see also [46]). In the context of this task, patients are presented with names of animals and are cued to make a button response regarding a particular attribute of the animal. This condition is alternated with a tone decision task where patients are presented with sequences of high- and low-frequency tones and are instructed to make a button response upon hearing a sequence containing two high tones.

For receptive language mapping with MEG, variations of the following task [47] have typically been used: patients are given a recognition memory task for spoken words, and Event Related Fields (ERFs) are recorded for each word stimulus. The stimuli (target words that are repeated and foils that are presented once) are delivered binaurally at the patient’s outer ear through two plastic tubes terminating in ear inserts with a variable interstimulus interval of 2.5–3.5 s. Patients are asked to lift their index finger whenever they recognize a repeated word. The responding hand is counterbalanced across sessions. On occasion, a variation of this protocol has been adopted in the visual modality, whereby target and distractor stimuli are presented visually, with identical task demands (e.g., [48]). As well as eliciting reliable receptive language-related activation, the visual variant of the task has been shown to engage the inferior frontal region (see [49]). Although MEG-receptive language mapping has most readily been achieved using the aforementioned protocol, the adoption of other paradigms (e.g., [50,51]) has been shown to be similarly useful in identifying the receptive language cortex. Expressive language mapping using MEG is typically performed in the context of a picture naming task (e.g., [52,53]) or of covert verb generation tasks (e.g., [50,54]).

### 2.3. The Reliability and Validity of Non-Invasive Methods

The use of the non-invasive fMRI and MEG as adjuncts to or as replacements of the invasive CSM and the Wada procedures in presurgical mapping, as well as their use in experimental investigations involving normal subjects, is justified by the fact that they provide compatible results with those of the invasive methods. The compatibility of the lateralization results of the Wada and the fMRI methods has been attested in a number of studies with patient samples ranging from 7 to 100 individuals. Results range from reporting perfect concordance [55,56,57] to nearly perfect [58,59,60,61,62] or considerably high [46,63,64,65,66,67,68].

Also high is the reported compatibility between the results of the Wada and MEG methods with respect to language lateralization, reaching 87% concordance in the study with the largest sample [47], with the rest of the studies reporting uniformly high agreement [48,50,54,69,70,71,72,73,74]. Far fewer studies report comparisons of laterality estimates for memory between fMRI and Wada. These involve small samples yet high concordance [75], but also low [76] and none between MEG and Wada.

Furthermore, the degree of concordance between the CSM and MEG localization of language-specific cortical patches is quite high. In a study involving a small patient sample, ref. [77] showed the compatibility of CSM and MEG for localizing receptive language-specific cortical sites, as did a second study involving 47 patients [78].

The question then arises as to how to interpret cases of discordant localization and lateralization results between the invasive and noninvasive methods. On the basis of the assumption that CSM and the Wada test are the gold standards, the tendency is to consider discordant estimates as failures of the noninvasive methods. However, when that assumption was put to empirical test, it became obvious that neither CSM results nor those of the Wada should be considered as the gold standard any more than the results of the noninvasive methods should. For example, using CSM, Ojemann [79,80] reported extensive temporal lobe involvement in receptive language tasks such as naming, yet Sanai et al. [81], also using CSM, found a paucity of naming sites there. In addition to limited reliability, CSM also has limited predictive value with respect to postsurgical language and memory performance when the latter is also operationally defined as performance in naming tasks. For example, Ojemann and Dodrill [82] reported an 80% predictive accuracy of CSM. Cervenka et al. [83] reported that in a series of seven patients, language deficits were not anticipated by CSM data because four had amygdalohippocampectomies and, more importantly, because three developed language deficits, although CSM-determined language-specific loci were not resected. Carvenka et al. [84] reported that three of four patients operated on presented language deficits that were not predicted by CSM; Hamberger et al. [85] reported that, in their experience, sparing cortical sites that were CSM-positive (i.e., their stimulation interrupted naming) did not prevent postoperative word finding difficulties; Hermann et al. [86], in their review of the results of eight centers involving 217 patients, concluded that neither intra- nor extraoperative CSM-guided surgeries are any more effective in reducing postoperative naming deficits than non-CSM-guided surgeries.

The efficacy of the Wada procedure is also lower than would be expected for a gold standard for predicting the likelihood of postoperative language and memory deficits. In fact, the assertion that it is correctly assessing language laterality was not empirically verified against surgical outcome except in the context of comparing its efficacy against that of fMRI. Such comparisons show that fMRI may in fact have better predictive efficacy than the Wada test [87].

For nearly two decades, MEG and fMRI data have been used as adjunct means of assessing language and memory laterality and language localization. In some cases, MEG especially has been used as a means of informing the placement of subdural grid electrodes [88,89,90] in the process of identifying the location and extent of the epileptogenic zones. The question, however, remains: can the noninvasive procedures used in tandem replace CSM and the Wada test? This issue has been debated for some time and until resolved, the invasive procedures will remain in many, but not necessarily in most clinicians’ minds the gold standards for the presurgical evaluation of language.

## 3. Applications in Basic Research

Whereas the clinical applications of imaging language-related networks aim at disclosing the areas not to be interfered with during surgery, the aim of basic neuroimaging research—which is a far more difficult to accomplish—is that of specifying what particular aspects of language perception and production the networks within each of these areas mediate.

### 3.1. Acoustic and Phonological Operations

In the following paragraphs I will summarize the functional neuroimaging findings that are germane to the predictions of the various models that have been alluded to previously regarding acoustic and phonological operations. Specifically, are the mechanisms of acoustic signal analysis located within the HG, and is the activation of HG bilaterally symmetrical, for both speech (syllables or words) and for non-speech sounds? Is the dominant model correct in stating that the phonological analysis of heard speech (whether of syllables, words or phrases) is meditated by a network located in the left pSTG, or are the alternative notions correct, predicting the activation of both the left and the right pSTG for this operation? Is the IFG also implicated in phoneme perception, as the “analysis by synthesis” theory of speech perception [91] implies?

It was mentioned in the introduction that it is generally asserted that the auditory analysis of non-speech sounds is mediated by the left and the right HG equally, whereas deriving the phonological or speech-specific features of sounds is mediated by distinct, left-hemisphere networks. In fact, there are two different hypotheses regarding the manner of neuronal mediation of the acoustic and phonological operations: the older hypothesis [92] is that whereas all acoustic operations extracting pitch, loudness, timbre, etc., from all sounds (both speech and non-speech) are bilaterally mediated, phonological features that are unique to speech sounds are extracted by a separate “speech organ” (i.e., a specialized neuronal network) located in the left hemisphere. The second hypothesis states that speech sounds are processed by the same networks as all other sounds, and if there is a hemispheric specialization it consists of the greater efficiency of left hemisphere networks in resolving fast temporal changes in the acoustic signals that characterize speech sounds and in the greater efficiency in right hemisphere networks in analyzing the spectral composition of all sounds (see [93]). According to this second hypothesis, there is no sharp separation between acoustic and phonological operations, and the spectral and temporal analysis of both speech and non-speech signals proceed bilaterally.

The validity of the latter hypothesis has been upheld in studies that have confirmed the specialization of the left hemisphere networks for detecting rapid temporal variations [94,95,96,97]. One PET study in particular [98] has demonstrated that whereas the left more than the right HG responds to temporal variation, the right anterior STG more than the left responds to spectral variation. Yet, in a subsequent fMRI study [99], it was found that high temporal variation in tonal stimuli was associated with bilaterally symmetrical HG activation and spectral variation was associated with bilaterally symmetrical STG activation and the activation of the posterior part of the superior temporal sulcus (STS). Moreover, in a study using electrocorticographic (ECoG) recordings, Hullett et al. [100] found that the spectro-temporal analysis of speech input engages large sectors of the STG of both hemispheres well beyond HG, with the pSTG specialized for fast temporal variations in the speech sounds and the anterior STG for slower such variations. Finally, a further complication in determining the manner of mediation of acoustic and phonological operations has arisen in an MEG study [101], the results of which challenge a long-standing assumption; namely, that phonological processing follows the acoustic analysis effected by HG, bilaterally. In this study, it was found that in the context of a speech perception task greater left HG activation, as well as more general left-lateralized activation, starts as early as 50 ms. for stimulus onset.

From two meta-analyses of neuroimaging studies of language [102,103], a somewhat different picture emerges with respect to the way acoustic and phonological operations are mediated. A distinctive aspect of that picture is the confirmation of the notion that the anterior and posterior language-related networks are co-activated during both production and comprehension tasks, an aspect that brings to mind the analysis by synthesis theory of speech perception [91]. Another is that, as far as neuroimaging data are concerned, language processing entails bilateral activation. Specifically, during speech perception tasks, whether listening to syllables [104] or producing speech sounds [105] or articulating phonemes [106] or engaging in rhyming word tasks [107] or attending selectively to vowels [108] or detecting temporal changes in vowels [109], but also during covert syllable repetition [110], HG, along with an anterior region overlapping with HG, was reliably activated bilaterally [103].

Also, bilateral activation was found in a region of the pSTG that includes the planum temporale while listening to vocalizations [111] and during the perception of syllables [112], the identification of syllables [104], while listening to syllables [113], while categorizing syllables [114], listening to pseudo-words [115], but also during word production [116], as reported by Vigneau et al. [103]. However, the same region also showed additional unilateral left hemisphere activation in the same tasks, thus rendering the question of whether phonological processes engage both the left and right pSTG difficult to answer. Does the preponderance of left pSTG activation mean that the observed right pSTG activation is superfluous? Or does it mean, as some believe, that although the right STG is capable of performing phonological analyses of speech in isolation as much as the left pSTG is, it is actively inhibited by the left?

The same questions are raised (and also remain unsettled) by activation observed mostly over Boca’s area, but also in its homotopic region of the right hemisphere. There, as well, activation attends both phonological production and perception tasks. For example, the left lateralized activation of the pars triangularis was found in tasks involving counting syllables [117] or identifying them [113], and between pars orbicularis and the middle frontal sulcus during syllable articulation tasks either overt [106] or covert [118], syllable counting [117], categorization [114], discrimination [113] and covert pseudo-word reading [119]. Similarly, the lower part of the pre-central gyrus is activated bilaterally (but, again, mostly in the left hemisphere) by the same phonological production and perception tasks, such as the overt articulation of words and syllables but, importantly, also during tongue movements, non-speech motor tasks involving the articulators [105,120], overt phoneme repetition [106], covert syllable repetition [110] and syllable counting [117].

Clearly, therefore, neuroimaging studies have raised more questions than they have resolved regarding detailed aspects of the cerebral mediation of acoustic and phonological operations—a phenomenon common to all branches of ever-evolving science—confirming, meanwhile, the notion that the phonological networks are lateralized to the left STG.

### 3.2. Semantic Operations

Networks of semantic operations are supposed to output not simply patterns of neuronal signals, but meaning as well, possibly by activating neuronal engrams of words or, possibly, because the signal patterns themselves somehow produce conscious experiences of meaning. The ambiguity of what it means for a pattern of neuronal signals to produce experiences either directly or indirectly aside, the lesion data summarized in the “Introduction” of this review appear to indicate that more than half of the left temporal and extensive sectors of the left frontal and parietal lobes are somehow implicated in that process. To the same conclusion point the results of early direct CSM studies of Penfield and his group [121] and of Ojemann and his associates [122]. Interestingly, the results of most functional neuroimaging studies summarized by Vigneau et al. [102,103] present the same picture.

The first obvious regularity evident in the neuroimaging data is that unlike phonological processing, semantic processing is more clearly lateralized in the left hemisphere. According to Vigneau et al.’s [103] meta-analysis, only 12.5% of activated sites were found in the right hemisphere during all word comprehension conditions reviewed, as opposed to 30% in the case of phonological processing. These sites fell mostly in the junction between the pars opercularis of the IFG and the middle frontal sulcus, in the anterior insula and the orbital region of the IFG. However, the activation of these right hemisphere sites was not specific to the semantic operations but also occurred in attentional tasks common to both phonological and semantic processing, as well as to working memory tasks (e.g., [123,124,125]).

In contrast, the activation of regions within the left frontal lobes does appear to be specific to the process of retrieving or creating word meaning. These regions lie anteriorly to the ones implicated in phonological processing on the opercular part of the IFG and at the junction of that region with the precentral gyrus (e.g., [126,127]) and in the orbital part of the IFG [128,129,130,131]. The manner in which these frontal regions contribute to the emergence of meaning and whether they do so in a different or complementary way to that of other areas also implicated in the same process, such as the ATL (see [36]), is not clear. What is sufficiently clear, however, is that the emergence of word meaning does appear to involve the anterior hub of the left lateralized language network and not only the posterior ones in the temporal and parietal regions or the right ATL.

In the Vigneau et al. [102] meta-analysis, several clusters of activation sites within the left temporal and parietal lobes were found in the posterior part of the superior temporal sulcus, the anterior part of the fusiform gyrus and the angular gyrus, but not in the pSTG region, against the predictions of the dominant model mentioned in the “Introduction”. The anterior superior temporal sulcus appears to be involved in the processing of written words, in that it is activated during written word categorization tasks [132,133,134,135,136] or in word reading [137,138,139,140,141], but its specific role remains conjectural, as is the precise role of the rest of the aforementioned activated regions. For example, the AG is said to be involved in conceptual knowledge [102] and the fusiform gyrus in word reading (see the subsequent section), but also in listening to words (e.g., [142]) and in word association tasks (e.g., [45,131,143,144]), but once again the neuroimaging evidence is insufficient to identify the precise role of these areas in the process of the emergence of word meaning. The same can be said for areas like the frontal pole (part of the left ATL), which are activated not only during semantic but also during syntactic tasks.

It can therefore be concluded that functional neuroimaging evidence supports the contention that, first, like phonological processing, the emergence word meaning also appears to require the involvement of the left frontal lobe. Second, that the contribution of the right hemisphere in the process is minimal and that the pSTG is not necessarily part of the process. But functional neuroimaging has yet to answer the question as to the precise role of each of the activated areas in the meaning-accessing or generation processes.

### 3.3. Syntactic Operations

Both the perception and the production of sentences are processes akin to the perception and production of words, in that they require the ordering of units (words and phonemes, respectively). One question, therefore, is whether the networks that mediate the ordering of phonemes are distinct and potentially distinguishable, through imaging, from those mediating the ordering of words. But the question is difficult to address for the following reason: to extract the activation pattern that is specific to the syntactic operations from the global activation pattern that also includes semantic, phonological and acoustic operations, one must contrast the activation pattern obtained during the processing of syntactically correct sentences with that obtained during processing of incorrect sentences. But what exactly the activation pattern resulting from that contrast corresponds to is not clear. It is certainly not necessary that it corresponds to the “syntax networks”, since in both cases such networks must be activated, in the first case resulting in the emergence of the meaning of the sentence, and in the second in the failure of their application to engender such meaning. It is therefore not surprising that most activation resulting from sentence processing studies is due to non-syntactic operations.

For example, in an early study [17] it was found that both the right and left ALTs were activated during listening to syntactically correct sentences. Although the implication would be that these regions are specific to syntactic operations or that they contain part of the (hypothesized) syntactic networks, it is by no means the correct one, given that these brain regions have also been found to be activated during a variety of tasks ranging from episodic memory retrieval (e.g., [145,146]) to word meaning retrieval (e.g., [147,148]).

The same ambiguity besets the interpretation of activation data in other parts of the temporal lobes in neuroimaging studies attempting to identify the “syntax” networks in the human brain. Progress in this area is likely to be made when alternatives (perhaps radically so) to the existing psycholinguistic models are formulated and tested.

### 3.4. Prosody

Prosody is used for two purposes: first to disambiguate the meaning of utterances (linguistic prosody), and second for conveying the affective state of the speaker. Although it would be reasonable to suggest that when the purpose of prosody is to disambiguate the meaning of utterances, left hemisphere networks are implicated, and when it conveys the affective state of the speaker right hemisphere ones are, no data have settled this issue.

The few studies that address the issue of linguistic prosody do not suffice for the drawing of definitive conclusions. The evidence regarding the mediation of affective prosody is equally provisional. The earliest study of affective prosody using electrophysiological estimates of laterality [149] showed that attending to the affective aspect of conversations conducted in a language unknown to the subjects engages predominantly the right hemisphere, whereas attending to phonetic aspects of the same conversation engages predominantly the left. Yet, subsequent investigations are less conclusive on this point. In a recent meta-analysis of twenty-seven neuroimaging studies of affective prosody by Witteman, Van Heuven and Schiller [150], the notion that prosodic processing involves the same fronto-temporal language networks and the notion that affective prosody is right-lateralized were confirmed. The studies analyzed fall into two categories: The first includes those in which affective prosody passages were contrasted with equivalent passages not carrying prosodic features. The second includes those studies in which the same prosodic passages were presented during two conditions, but the subjects’ one task was to identity or distinguish the emotions expressed by the prosody in the one condition and in the other to identify other non-affective features of the same passages.

In both sets of studies, once again, a frontal-temporal network was activated involving STG and ITG sites. Moreover, in the first set of studies (but not the second), the left and right medial frontal gyrus and the left and right insula were activated, possibly reflecting the affective state perceived or the affective state induced by the material in the hearers. With respect to lateralization, the expected right hemisphere preponderance for affective prosody involved only the transverse temporal gyrus in the first set of studies and the pSTG in the second, neither of which structures are known to be involved in the perception of affect, thus leaving the issue of the lateralization of affective prosody unsettled.

### 3.5. Sign Language

Unlike so-called “body language”, which, among oral language users, serves to express the attitude of the speaker, sign languages have many of the purely linguistic features that also characterize oral languages. For example, there is a correspondence between phonemes and particular finger movements named “cheremes” (from the Greek for hand: cheri). There is also a correspondence between words and “signs” consisting of one or more cheremes [151]. There is finally correspondence between speech prosody, both linguistic and affective, and distinct facial expressions as well as body movements used for the same purposes by sign language users. It is, therefore, expected that there might be an overlap of networks mediating oral language and sign languages. Accordingly, the main question to be addressed here is whether neuroimaging data also accord with that expectation and whether they better define the constituent hubs or nodes of the presumed left lateralized sign language network.

The earliest of the neuroimaging studies to address this issue was that of Neville and her associates [152]. In that, as well as in subsequent similar studies, the following findings emerged: first, left-lateralized language networks activated, corresponding approximately to Brodman’s areas (BA) 44/45 within the IFG (i.e., Broca’s area) and to BA 22, roughly corresponding to part of Wernicke’s area but also extending over the entire STG, when English speakers were reading English sentences and when native American Sign Language (ASL) users were perceiving signed sentences. Second, congenitally deaf ASL signers showed activation of right hemisphere regions homotopic to those of the oral language network. This effect, however, does not characterize bilingual signers who have been exposed to oral language, who showed bilateral activation of the receptive language cortex but engaged the left anterior regions typically associated with speech production in order to comprehend the meaning of ASL sentences—a finding that argues in favor of the notion that regardless of whether the task requires comprehension or production, the language network is activated as a unit.

Several other studies of the comprehension of signed sentences have addressed the hypothesis of a common network at the basis of sign and oral languages. Newman and his associates [153] exposed native signers and hearing English speakers to “signed” and English sentences alternating with control stimuli that were visually similar to the linguistic stimuli but meaningless. This fMRI study supported the expected left-lateralized activation pattern, and showed that both native and late signers (i.e., individuals that had learned to sign later in life) engaged the left-lateralized network, including Broca’s region, the dorsolateral prefrontal cortex, the temporal sulcus, both left and right superior temporal sulcus and the angular gyrus (AG), during signed sentence comprehension. A third group of late signers exhibited an essentially similar activation pattern. Also, contrary to the right dominant activation pattern found in Neville et al.’s [151] study and in concert with the Newmann et al. [152] findings, Sakai and his associates [154], in an fMRI study of deaf signers of the Japanese Sign Language (JSL), also found a left-lateralized network for signed sentences (dialogues) comprehension featuring the IFG, mid-frontal and dorsolateral cortex, the MTG (but not the STG), the SMG and the AG.

Subsequently, Newmann and associates [155,156], also using fMRI during sentence comprehension with deaf ASL signers, replicated the largely left anterior cortex activation (pars triangularis or BA 45 of the IFG) but failed to replicate the bilateral activation of the middle part of the MTG, the superior temporal sulcus (STS) and the left AG. The same group [157] addressed the issue of whether ALS and meaningful yet not linguistic gestures would activate the same left-lateralized network, and concluded that symbolic gestures are indeed processed by the left lateralized network, indicating that they are treated as if they were linguistic in nature.

### 3.6. Reading and Dyslexia

Although clinical observations have indicated that certain focal lesions result in alexia, the inability to read, they have not offered any indications regarding the networks necessary for reading, or what changes in these networks may account for dyslexia when the latter is not associated with lesions. Normal reading appears to be accomplished in three stages, each of which entails its own neuronal network. The first stage, common to all visual perception, is mediated by the visual cortex; the second is peculiar to written words or to word-like stimuli. It entails one of two distinct yet compatible operations. The one operation is known as the “grapheme–phoneme rule system”. It is supposed to convert the output of the primary visual analysis of letter shapes to their sound equivalent, and its neuronal basis was postulated as early as 1892 by Dejerine [158] to be contained in the left angular gyrus [159]. The output of this conversion operation would then access the language production and perception system and the written word would thus be read and understood (or understood and read).

The second operation is known as the “visual word form system” [160] and its network is supposed to be located in the left fusiform gyrus at the base of the temporal lobe (e.g., [161,162,163]). The output of this network then accesses the semantic comprehension and the production networks by directly activating the circuits that code the meaning that corresponds to the word form, and thus the words are read and understood (or, again, understood and read). These two operations are not mutually exclusive. In fact, they may either both belong to the second stage of the reading process (the third stage being the engagement of the language comprehension and production system), or the visual word form system may be engaged first, sending its output to both the grapheme-to-phoneme conversion mechanism and to the semantic language comprehension system.

The temporal succession of the activation of these networks is beyond the range of either fMRI or the PET in that it unfolds within milliseconds from the arrival of the visual stimuli at the primary visual cortex. It is, however, detectable through magnetoencephalography (MEG). In fact, a series of MEG investigations [164,165,166,167,168,169] provided evidence showing that following bilateral visual cortex activation in response to word or pseudoword stimuli, left lateralized basal temporal activation precedes the engagement of the left posterior and anterior language networks, which are activated about 300 milliseconds following the activation of the primary visual cortex.

A meta-analysis of reading studies [160] provided further indications that the early stage of the reading process engages the left occipitotemporal region in the vicinity of the fusiform gyrus, that is, the word form area, a finding that agrees with the aforementioned MEG results as well as the results of subsequent investigations (e.g., [163,170]). The same meta-analysis also provided indications that components of the grapheme-to-phoneme conversion process are most likely located in the left posterior STG, the supramarginal gyrus (SMG), but also in the pars opercularis part of Broca’s area (BA 44), a finding also supported by more recent investigations [170]. Subsequent meta-analyses [171,172] have resulted in similar findings. The former provides additional evidence for a grapheme-to-phoneme conversion network in the inferior parietal region, and the latter evidence of reading-related activation in the ventral aspect of the left occipto-temporal and inferior parietal region, in addition to the IFG.

Given that lesions in the left fusiform “word form area”, as well as in the vicinity of Wernicke’s area, result in alexia (among other symptoms), the question naturally arises whether dyslexia, not associated with structural lesions, is due to a malfunction of either of these two areas. The data appear to favor this explanation. For example, in an MEG study, Simos et al. [168] showed that unlike normally reading children who engaged the pSTG region, children diagnosed with dyslexia failed to engage that region but engaged the homotopic area in the right hemisphere, instead. Moreover, after successful behavioral reading intervention, the same children displayed the normal activation profile during reading. Similar findings have appeared in subsequent years. Evidence from two meta-analyses [173,174] showed that the main difference between normal and dyslexic readers was the suppressed activation of the left lateralized language network among the latter. In addition, in another meta-analysis of 13 fMRI and PET studies of normal readers and individuals with reading difficulties, Pollack and associates [175] found that during rhyming or reading tasks dyslexics engaged to a greater degree the right rather than left hemisphere structures. These findings point to the possibility that dyslexia is due to a malfunctioning of articulatory and “word form” analysis mechanisms rather than the mechanism of grapheme to phoneme conversion.

Once again, although functional neuroimaging studies generate many more questions regarding the way neuronal networks engender language than they set out to answer, they do, to a considerable degree, verify most of the explanations regarding the neurophysiology of language, in all its forms, that has been and continues to be generated in the clinic through the observation and the interpretation of the effects of focal lesions. It is in part due to this convergence of the two sets of relevant evidence that non-invasive presurgical brain mapping has become a reality.

## 4. Perspectives

This review includes studies of mostly European languages and Japanese; therefore, the results may not be readily generalized to the cerebral mediation of tonal languages like the Chinese that may possibly involve different neuronal networks, especially for phonological processing (e.g., [176]). Also, some studies involve tasks of questionable ecological validity, whereas others may not sample satisfactorily all aspects of meaningful communication (e.g., non-verbal semantics). Such shortcomings, driven largely by the practical constraints that functional neuroimaging procedures entail, especially in the pre-surgical brain mapping of patients, are appreciated and will be overcome, in all likelihood, in the future. But for this to happen in an efficient way, future research should aim not only at the procurement of new results, as is now the case, but also be directed to the consolidation of old findings through replication within and across laboratories. Clearly, for this to happen, a change in opinion as to what constitutes commendable research activity is required on the part of both researchers and funding institutions. Such changes do not transpire easily but are absolutely necessary in weeding out inadvertent false findings that survive unchecked for years in the guise of facts—a phenomenon rather common in functional neuroimaging, as well as in many other fields of science.
